# Analyzing the outcome of centripetal and coriolis acceleration on a nonlocal thermoelastic material using the modified Green-Lindsay hypothesis

**DOI:** 10.1038/s41598-026-67145-1

**Published:** 2026-08-28

**Authors:** Samia M. Said

**Affiliations:** Department of Mathematics, Faculty of Science, Zagazig University, P.O. Box 44519, Zagazig, Egypt

**Keywords:** Modified Green-Lindsay theory, Nonlocal thermoelastic solid, Rotation, Temperature-dependent properties, Engineering, Materials science, Mathematics and computing

## Abstract

This study investigates the influence of rotation on a nonlocal thermoelastic material with temperature-dependent properties using the modified Green–Lindsay framework of generalized thermoelasticity. Exact analytical expressions for the displacement, stress, and temperature are obtained by employing the normal mode analysis without imposing simplifying assumptions on the field variables. Numerical computations are performed using MATLAB R2013a under appropriate boundary conditions. The results reveal that increasing the rotation parameter significantly alters the propagation and amplitude of thermoelastic waves, while the nonlocality parameter reduces the peak stress and temperature distributions by approximately 45% and 25%, respectively. Furthermore, variations in the empirical material coefficient produce noticeable changes in the displacement and stress responses, confirming its important role in the thermoelastic behavior of the medium. Compared with the corresponding reference model, the present temperature-dependent nonlocal formulation provides more realistic predictions of the coupled thermoelastic response, particularly in the presence of rotational effects. These findings are relevant to the design and analysis of advanced engineering materials used in aerospace structures, refractory components, and laser-assisted technologies.

## Introduction

Two fundamental discrepancies among the predictions of the uncoupled thermoelasticity framework and empirical observations necessitated further theoretical advancements. Firstly, despite the fact that elastic changes inherently produce thermal impacts, no elastic variables are included in the traditional heat conduction relation. Secondly, the parabolic nature of the heat equation implies that heat waves propagate at infinite speeds, a physically unrealistic outcome. To address these inconsistencies, Biot^1^ introduced the coupled thermoelasticity (CD) hypothesis, which integrates thermal and elastic behaviors. Recognizing the need for further refinement, Hetnarski and Ignaczak^2^ reviewed various generalizations of coupled thermoelasticity. Among these, Lord and Schulman^3^ developed the generalized thermoelasticity (L-S) hypothesis incorporating a single thermal relaxation time. This framework is built on the Maxwell–Cattaneo^4^ modification of Fourier's heat conduction law, introducing a time delay to heat flux and overcoming the limitation of infinite propagation speeds. Building on this foundation, Green and Lindsay^5^ proposed an alternative generalized thermoelasticity hypothesis (G-L) that introduced two thermal relaxation times. This new formulation modified both the energy equation and the relations of motion, addressing prior limitations and extending thermoelastic analysis. More recently, Yu et al.^6^, applying principles of extended thermodynamics, formulated the modified Green-Lindsay (MGL) framework of thermoelasticity by incorporating the strain rate term into Green-Lindsay's original hypothesis. Collectively, these hypotheses known as generalized thermoelasticity frameworks—resolve the issue of heat propagation at unlimited velocities. Subsequent researchers have studied and applied these generalized frameworks to a variety of complex scenarios. The modified Green-Lindsay (MGL) hypothesis represents a sophisticated approach within generalized thermoelasticity. It incorporates thermal relaxation times and accounts for strain-rate effects in both heat conduction and elasticity equations. This framework offers a significant advantage by delivering physically realistic models for extreme thermal shocks, eliminating the non-physical discontinuities and infinite wave speeds often associated with traditional theories. The MGL model enables engineers to more accurately understand how materials deform in real-time under conditions of rapid heating, thereby reducing the risk of unexpected failures during practical applications. Quintanilla^7^ explored qualitative aspects of the MGL framework, while Sarkar et al.^8^ displayed the reflection and propagation of thermoelastic harmonic waves in a thermoelastic half-space medium using the MGL framework. Kalkal et al.^9^ extended these applications by studying plane wave spread in fiber-reinforced rotating thermoelastic material using the MGL hypothesis. Shivay and Mukhopadhyay^10^ advanced the understanding of thermoelastic interactions by investigating functionally graded hollow disks through the lens of the modified Green-Lindsay framework. Shakeriaski et al.^11^ contributed a nonlinear numerical technique to solve generalized thermoelastic fundamental equations in situations involving large deformations under thermal shocks, employing the MGL model for validation. Furthermore, Mirparizi and Razavinasab^12^ presented a description of electro-magneto-elastic functionally graded materials via the MGL hypothesis. Othman et al.^13^ addressed a two-dimensional problem in a nonlocal fiber-reinforced thermo-visco-elastic material under rotational effect, employing a comparative framework involving the MGL, G-L, and L-S theories to analyze their behavior comprehensively. Li et al.^14^ conducted an investigation into the effects of strain rate and size-dependent phenomena on the thermomechanical response of porous media. Their study proposed the development of a nonlocal poro-thermoelastic model via the model of the modified Green-Lindsay theory. This body of research demonstrates how thermoelasticity theories have evolved to address previously unresolved physical phenomena and continue to expand their applicability across diverse material systems and dynamic contexts.

Examining the propagation of planar thermoelastic waves in a rotating material appears more realistic, as large celestial bodies like the Earth, the Moon, and other planets exhibit angular motion (i.e., they are rotating bodies). This area of study is particularly important for predicting material stability in environments involving high-speed rotation, such as aerospace components, robotics, and planetary geology. In these scenarios, Coriolis and centrifugal forces significantly influence temperature distribution and the behavior of wave propagation. Several researchers have explored various aspects of thermoelastic behavior in such rotating media, including Schoenberg and Censor^15^, Puri^16^, Choudhuri and Debnath^17^, Abd-Alla and Mahmoud^18^, Said et al.^19,20^, Kadian et al.^21^, Nazir and Kumar^22^, Ramady et al.^23^, and Abouelregal et al.^24,25^.

Nonlocality in a thermoelastic medium refers to the phenomenon where the response at a given point is affected by deformations occurring in surrounding regions, extending beyond its immediate vicinity. The introduction of a nonlocal parameter alters the stress, displacement, and temperature distributions, frequently resulting in oscillatory patterns and intricate interdependencies. Eringen^26^ was the pioneer in proposing the nonlocal elastic hypothesis. Over a span of two years, Eringen^27^ explored the concept of nonlocal thermoelasticity in depth. His work involved examining constitutive formulations, governing equations, equilibrium principles, and the relationships between displacements and temperatures within the framework of nonlocal elastic theory. The influence of the nonlocal parameter on different thermoelastic media has been extensively examined in various studies and articles, covering a broad spectrum of topics^28–34^.

The analysis of a rotating nonlocal, isotropic thermoelastic material within a half-space explores complex thermomechanical phenomena influenced by microstructural length scales and rotational forces. This serves as a robust theoretical framework for advancements in aerospace design, seismology, and microscale engineering applications. This study explores the outcome of rotation and temperature-dependent properties on a nonlocal thermoelastic material within the framework of the MGL framework. The governing equations, rendered in a dimensionless form, are solved through normal mode analysis to evaluate the temperature distribution, displacement components, and stress fields. Results are analyzed by comparing the impacts of varying rotation rates and locality parameter values. Additionally, comparisons were conducted for variable values of the empirical material coefficient to assess their impact. The findings highlight the substantial impact of polymer microstructure and thermal properties on small-scale dynamics, providing meaningful parametric insights.

## Mathematical formulation and the fundamental relations

**Fig. 1:**
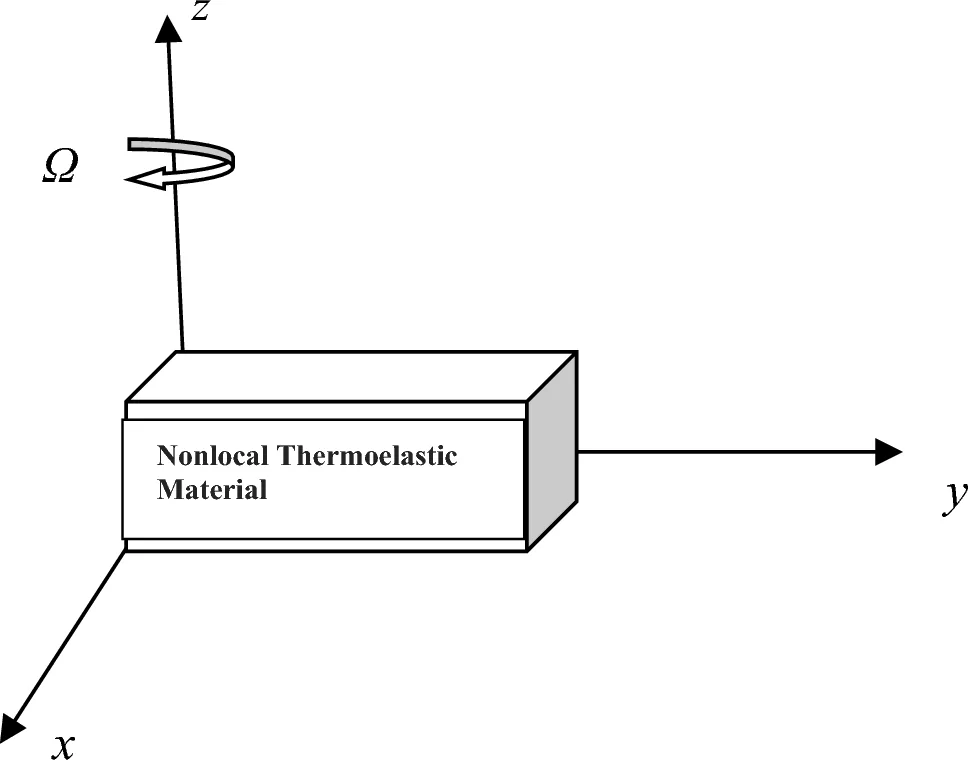
The sketch of the problem.

We consider a rotating nonlocal homogeneous thermoelastic isotropic material in a half-space $(\,x \ge 0\,)$ using a modified Green-Lindsay framework with temperature-dependent properties. Since the medium is rotating uniformly with an angular velocity $\underline{\Omega } \,\, = \,\,(\,0,\,0,\,\,\Omega )\,,$ the displacement equation of motion in the rotating frame of reference has two additional terms (Schoenberg and Censor^15^): centripetal acceleration, and Coriolis acceleration as in Fig. 1.

A plane strain in the *xy* plane with the displacement vector

1 $$\underline{u} = (u_{1} ,\,u_{2} ,0)\,,\,\,\,\,\,\,\,\,\,\,\,\,\,\,u_{1} = (x,y,t),\,\,\,\,\,\,\,u_{2} = (x,y,t)\,.$$

The stress–strain relations are as in Eringen^27^.

2 $$\left( {1 - \varepsilon^{2} \,\nabla^{2} } \right)\,\,\,\sigma_{ij} = (1 + \tau_{1} \frac{\partial }{\partial \,t})\,\left[ {\lambda \,\varepsilon_{kk} \delta_{ij} + 2 \mu \,\varepsilon_{ij} - \gamma \,\,\theta \,\delta_{ij\,} } \right]\,\,.$$

The equations of motion with the impact of rotation as Schoenberg and Censor^15^

3 $$\rho \,\,\left[ {\frac{{\partial^{2} \,u_{i} }}{{\partial t^{2} }} + (\underline{\Omega } \wedge (\,\underline{\Omega } \wedge \underline{u} \,))_{i} + 2(\,\underline{\Omega } \wedge \underline{{\dot{u}}} \,)_{i} } \right] = \sigma_{ji,j} \,.$$

The equation of heat conduction via the MGL model as Yu et al^6^.

4 $$K\,\,\nabla^{2} \theta = \left( {1\, + \tau_{0} \frac{\partial }{\partial t}} \right)\,\,\left( {\rho \,C_{E} \,\dot{\theta } + \,\,\gamma \,\,T_{0} \,\dot{e}} \right)\,.$$

We take the following assumption as Said^35^:

5 $$\mu = \mu_{0} \left( {1 - \delta T_{0} } \right),\,\lambda = \lambda_{0} \left( {1 - \delta T_{0} } \right),\,\gamma = \gamma_{0} \left( {1 - \delta T_{0} } \right)$$

In the absence of the temperature-dependent properties, the modulus of elasticity *δ = 0*.

Introducing Eqs. (2) in Eqs. (3), thus we have

6 $$\,(1 - \varepsilon^{2} \nabla^{2} )\,\,\left( {\ddot{u}_{1} - \Omega^{2} u_{1} - 2\Omega \,\dot{u}_{2} } \right) = \,(1 - \delta \,T_{0} )\,\,(1 + \tau_{1} \frac{\partial }{\partial \,t})\,\,\left( {(\lambda_{0} + 2\mu_{0} )\,\frac{{\partial \,u_{1} }}{{\partial \,x^{2} }} + (\lambda_{0} + \mu_{0} )\,\frac{{\partial \,u_{2} }}{\partial \,x\,\partial \,y} + \mu_{0} \frac{{\partial \,u_{1} }}{{\partial \,y^{2} }} - \gamma_{0} \,\frac{\partial \,\theta }{{\partial \,x}}} \right)\,\,,$$

7 $$\,(1 - \varepsilon^{2} \nabla^{2} )\,\,\left( {\ddot{u}_{2} - \Omega^{2} u_{2} + 2\Omega \,\dot{u}_{1} } \right) = \,(1 - \delta \,T_{0} )\,\,(1 + \tau_{1} \frac{\partial }{\partial \,t})\,\,\left( {\mu_{0} \frac{{\partial \,u_{2} }}{{\partial \,x^{2} }} + (\lambda_{0} + \mu_{0} )\,\frac{{\partial \,u_{1} }}{\partial \,x\,\partial \,y} + (\lambda_{0} + 2\mu_{0} )\,\frac{{\partial \,u_{2} }}{{\partial \,y^{2} }} - \gamma_{0} \,\frac{\partial \,\theta }{{\partial y}}} \right)\,\,.$$

The next non-dimensional quantities are presented for convenience:

8 $$\begin{aligned} (x^{\prime } ,y^{\prime } ,\varepsilon ^{\prime } ,u_{1}^{\prime } ,u_{2}^{\prime } ) = & \frac{1}{{l_{0} }}(x,y,\varepsilon ,u_{1} ,u_{2} ),\;(t^{\prime } ,\tau _{0}^{\prime } ,\tau _{1}^{\prime } ) = \frac{{c_{0} }}{{l_{0} }}(t,\tau _{0} ,\tau _{1} ),\theta ^{\prime } = \frac{{\gamma \theta }}{{\lambda + 2\mu }},\;\sigma _{{ij^{\prime } }}^{\prime } = \frac{{\sigma _{{ij}} }}{\mu },\; \\ \,\Omega ^{\prime } = \frac{{l_{0} }}{{c_{0} }}\Omega ,\;l_{0} = \sqrt {\frac{{K^{*} }}{{\rho C_{E} T_{0} }}} ,\;c_{0} = \sqrt {\frac{{\lambda + 2\mu }}{\rho }} \\ \end{aligned}$$

The governing equations above reduce to terms of the non-dimensional quantities described in Eq. (8) (dropping the dashes for simplicity)

9 $$\,(1 - \varepsilon^{2} \nabla^{2} )\,\,\left( {\ddot{u}_{1} - \Omega^{2} u_{1} - 2\Omega \,\dot{u}_{2} } \right) = \,\,\,(1 + \tau_{1} \frac{\partial }{\partial \,t})\,\,\left( {\,\frac{{\partial \,u_{1} }}{{\partial \,x^{2} }} + S_{1} \,\frac{{\partial \,u_{2} }}{\partial \,x\,\partial \,y} + S_{2} \,\frac{{\partial \,u_{1} }}{{\partial \,y^{2} }} - \,\frac{\partial \,\theta }{{\partial \,x}}} \right)\,\,,$$

10 $$\,(1 - \varepsilon^{2} \nabla^{2} )\,\,\left( {\ddot{u}_{2} - \Omega^{2} u_{2} + 2\Omega \,\dot{u}_{1} } \right) = \,\,(1 + \tau_{1} \frac{\partial }{\partial \,t})\,\,\left( {S_{2} \frac{{\partial \,u_{2} }}{{\partial \,x^{2} }} + S_{1} \frac{{\partial \,u_{1} }}{\partial \,x\,\partial \,y} + \frac{{\partial \,u_{2} }}{{\partial \,y^{2} }} - \,\frac{\partial \,\theta }{{\partial y}}} \right)\,\,,$$

11 $$\,\,\nabla^{2} \theta = \,\left( {1\, + \tau_{0} \frac{\partial }{\partial t}} \right)\,\,\left( {S_{3} \,\,\dot{\theta } + S_{4} \,\,\dot{e}} \right)\,,$$

where, $$S_{1} = \frac{{(\lambda_{0} + \mu_{0} )\,\,(1 - \delta \,T_{0} )}}{{\rho \,c_{0}^{2} }},$$ $$S_{2} = \frac{{\mu_{0} \,\,(1 - \delta \,T_{0} )}}{{\rho \,c_{0}^{2} }},$$ $$S_{3} = \frac{{\rho \,C_{E} \,c_{0} \,l_{0} }}{k},$$ $$S_{4} = \frac{{\gamma^{2} T_{0} c_{0} l_{0} }}{{k\,\,(\lambda_{0} + 2\mu_{0} )\,\,(1 - \delta \,T_{0} )}}.$$

## Normal mode analysis method

We carry out the problem of a rotating nonlocal thermoelastic material via normal mode analysis as:

12 $$\left( {\,u_{1} ,u_{2} ,\,\,\theta ,\,\,\sigma_{ij} \,} \right)\,\,(x,y,t) = \left( {\,u_{1}^{*} ,u_{2}^{*} ,\,\theta^{*} ,\,\,\,\sigma_{ij}^{*} \,\,} \right)\,\,(x)\,\,e\,\,\,^{{\left( {{\mathrm{i}}\,\,a\,y - mt} \right)}} \,,$$

here $u_{1}^{*} (x),$ etc. is the amplitude of the $u_{1} \,(x,y,\,t)$, etc.

Using Eqs. (12) in Eqs. (9)- (10), we get

13 $$\,(L_{1} D^{2} - L_{2} )\,u_{1}^{*} + \,(L_{3} D^{2} + L_{4} D - L_{6} )\,u_{2}^{*} - L_{5} D\,\theta^{*} \, = 0\,,$$

14 $$\,\,( - L_{3} D^{2} + L_{4} D + L_{6} )\,u_{1}^{*} + (L_{7} D^{2} - L_{8} )\,u_{2}^{*} - {\mathrm{i}}\,a\,L_{5} \,\theta^{*} \, = 0\,,$$

15 $$\,\,L_{9} \,D\,u_{1}^{*} + {\mathrm{i}}\,a\,L_{9} u_{2}^{*} + (D^{2} + \,L_{10} )\,\theta^{*} \, = 0\,,$$

where, $$L_{1} = \,L_{5} + \varepsilon^{2} (m^{2} - \Omega^{2} ),\,$$ $$L_{2} = a^{2} \,A_{2} L_{5} + (1 + a^{2} \varepsilon^{2} )(m^{2} - \Omega^{2} ),\,$$ $$L_{3} = \,2\,m\,\Omega \,\varepsilon^{2} ,\,$$ $$L_{4} = {\mathrm{i}}\,a\,A_{1} L_{5} ,\,$$ $$L_{5} = \,1 - \tau_{1} \,m\,,\,$$ $$L_{6} = 2\Omega m\,(1 + \varepsilon^{2} a^{2} ),\,$$ $$L_{7} = \,A_{2} L_{5} + \varepsilon^{2} \,(m^{2} - \Omega^{2} ),\,$$ $$L_{8} = a^{2} L_{5} + (1 + a^{2} \varepsilon^{2} )(m^{2} - \Omega^{2} ),\,$$ $$L_{9} = A_{4} m\,(\,1 - \tau_{0} \,)\,,\,$$ $$L_{10} = A_{3} m\,(\,1 - \tau_{0} \,m) - a^{2} \,,\,$$ $$\,{\mathrm{D}} = \frac{{\mathrm{d}}}{{{\mathrm{d}}x}}.$$

Removing $u_{2}^{*} (x)$ and $\,\theta^{*} (x)$ among Eqs. (13)- (14), the next equation satisfied by $u_{1}^{*} (x),\,u_{2}^{*} (x),\,\,\theta^{*} (x)$ can be acquired:

16 $$\left( {{\mathrm{D}}^{{6}} - G_{1} {\text{ D}}^{{4}} + G_{2} {\text{ D}}^{{2}} - G_{3} \, } \right) \, \left\{ {\,u_{1}^{*} {(}x{),}\,u_{2}^{*} {(}x{),}\,\theta^{*} {(}x{)}\,} \right\} = 0,$$

where,$$G_{1} = \frac{1}{{L_{1} L_{7} + L_{3}^{2} }}(L_{1} L_{8} + L_{2} L_{7} + L_{4}^{2} + 2L_{3} L_{6} - L_{1} L_{7} L_{10} - L_{3}^{2} L_{10} - \,L_{5} L_{7} L_{9} ),$$

$$G_{2} = \frac{1}{{L_{1} L_{7} + L_{3}^{2} }}(L_{2} L_{8} + L_{6}^{2} + L_{3} L_{6} L_{10} - L_{1} L_{8} L_{10} - \,a^{2} L_{1} L_{5} L_{9} - L_{2} L_{7} L_{10} - L_{5} L_{9} L_{8} - L_{4}^{2} L_{10} - L_{3} L_{6} L_{10} - 2{\mathrm{i}}aL_{4} L_{5} L_{9} ),$$

$$G_{3} = \frac{ - 1}{{L_{1} L_{7} + L_{3}^{2} }}(L_{2} L_{8} L_{10} + a^{2} L_{2} L_{5} L_{9} + L_{6}^{2} L_{10} )\,.$$

Equation (16) can be factored as

17 $$\left( {{\text{ D}}^{{2}} - \chi_{1}^{2} \, } \right) \, \left( {{\text{ D}}^{{2}} - \chi_{2}^{2} \, } \right)\,\,\left( {{\text{ D}}^{{2}} - \chi_{3}^{2} \, } \right)\,\,\,u_{1}^{*} (x) = 0,$$

where $\chi_{j}^{2} \,(\,j = 1,\,2,\,3\,)$ are the roots of the characteristic equation

18 $$\chi^{6} - G_{1} \chi^{4} + G_{2} \chi^{2} - G_{3} = 0\,.$$

The solution of Eq. (16), bounded as $x \to \infty ,$ can be expressed as:

19 $$u_{1}^{*} (x) = \pi_{1} \,{\mathrm{e}}\,^{{\,{(} - \chi_{1} x{)}}} + \pi_{2} \,{\mathrm{e}}\,^{{\,{(} - \chi_{2} x{)}}} + \pi_{3} \,{\mathrm{e}}\,^{{\,{(} - \chi_{3} x{)}}} {,}$$

20 $$u_{2}^{*} (x) = F_{11} \pi_{1} \,{\mathrm{e}}\,^{{\,{(} - \chi_{1} x{)}}} + F_{12} \pi_{2} \,{\mathrm{e}}\,^{{\,{(} - \chi_{2} x{)}}} + F_{13} \pi_{3} \,{\mathrm{e}}\,^{{\,{(} - \chi_{3} x{)}}} {,}$$

21 $$\theta^{*} (x) = F_{21} \pi_{1} \,{\mathrm{e}}\,^{{\,{(} - \chi_{1} x{)}}} + F_{22} \pi_{2} \,{\mathrm{e}}\,^{{\,{(} - \chi_{2} x{)}}} + F_{23} \pi_{3} \,{\mathrm{e}}\,^{{\,{(} - \chi_{3} x{)}}} \,{.}$$

Using Eqs. (8) and (12) in Eq. (2) and then using Eqs. (19) - (20), we get

22 $$\sigma_{xx}^{*} (x) = F_{31} \pi_{1} \,{\mathrm{e}}\,^{{\,{(} - \chi_{1} x{)}}} + F_{32} \pi_{2} \,{\mathrm{e}}\,^{{\,{(} - \chi_{2} x{)}}} + F_{33} \pi_{3} \,{\mathrm{e}}\,^{{\,{(} - \chi_{3} x{)}}} {,}$$

23 $$\sigma_{xy}^{*} (x) = F_{41} \pi_{1} \,{\mathrm{e}}\,^{{\,{(} - \chi_{1} x{)}}} + F_{42} \pi_{2} \,{\mathrm{e}}\,^{{\,{(} - \chi_{2} x{)}}} + F_{43} \pi_{3} \,{\mathrm{e}}\,^{{\,{(} - \chi_{3} x{)}}} {,}$$

where,

$$F_{11} = \frac{{L_{3} \chi_{1}^{3} - (iaL_{1} - L_{4} )\chi_{1}^{2} - L_{6} \chi_{1} + iaL_{2} }}{{L_{7} \chi_{1}^{3} + iaL_{3} \chi_{1}^{2} - (iaL_{4} + L_{4} )\chi_{1} - iaL_{6} }},\;F_{12} = \frac{{L_{3} \chi_{2}^{3} - (iaL_{1} - L_{4} )\chi_{2}^{2} - L_{6} \chi_{2} + iaL_{2} }}{{L_{7} \chi_{2}^{3} + iaL_{3} \chi_{2}^{2} - (iaL_{4} + L_{4} )\chi_{2} - iaL_{6} }},$$

$$F_{13} = \frac{{L_{3} \chi_{3}^{3} - (iaL_{1} - L_{4} )\chi_{3}^{2} - L_{6} \chi_{3} + iaL_{2} }}{{L_{7} \chi_{3}^{3} + iaL_{3} \chi_{3}^{2} - (iaL_{4} + L_{4} )\chi_{3} - iaL_{6} }},\;F_{21} = \frac{{L_{1} \chi_{1}^{2} - L_{2} + (L_{3} \chi_{1}^{2} - L_{4} \chi_{1} - L_{6} )F_{11} }}{{ - L_{5} \chi_{1} }},$$

$$F_{22} = \frac{{L_{1} k_{2}^{2} - L_{2} + (L_{3} \chi_{2}^{2} - L_{4} \chi_{2} - L_{6} )F_{12} }}{{ - L_{5} \chi_{2} }},\;F_{23} = \frac{{L_{1} k_{3}^{2} - L_{2} + (L_{3} \chi_{3}^{2} - L_{4} \chi_{3} - L_{6} )F_{13} }}{{ - L_{5} \chi_{3} }},$$

$$F_{31} = \frac{{(1 - \tau_{1} m)( - (\lambda_{0} + 2\mu_{0} )\chi_{1} + ia\lambda_{0} F_{11} - (\lambda_{0} + 2\mu_{0} )F_{21} )}}{{\mu_{0} (1 + a^{2} \varepsilon^{2} - \chi_{1}^{2} \varepsilon^{2} )}},\;F_{32} = \frac{{(1 - \tau_{1} m)( - (\lambda_{0} + 2\mu_{0} )\chi_{2} + ia\lambda_{0} F_{12} - (\lambda_{0} + 2\mu_{0} )F_{22} )}}{{\mu_{0} (1 + a^{2} \varepsilon^{2} - \chi_{2}^{2} \varepsilon^{2} )}}$$

$$F_{33} = \frac{{(1 - \tau_{1} m)\left( { - (\lambda_{0} + 2\mu_{0} )\chi_{3} + ia\lambda_{0} F_{13} - (\lambda_{0} + 2\mu_{0} )F_{23} } \right)}}{{\mu_{0} (1 + a^{2} \varepsilon^{2} - \chi_{3}^{2} \varepsilon^{2} )}},\;F_{41} = \frac{{(1 - \tau_{1} m)(ia - \chi_{1} F_{11} )}}{{1 + a^{2} \varepsilon^{2} - \chi_{1}^{2} \varepsilon^{2} }},\;F_{42} = \frac{{(1 - \tau_{1} m)(ia - \chi_{2} F_{12} )}}{{1 + a^{2} \varepsilon^{2} - \chi_{2}^{2} \varepsilon^{2} }},$$

$$\;F_{43} = \frac{{(1 - \tau_{1} m)(ia - \chi_{3} F_{13} )}}{{1 + a^{2} \varepsilon^{2} - \chi_{3}^{2} \varepsilon^{2} }}.$$

## Boundary conditions

In the present work, a nonlocal thermoelastic rotating solid half-space is taken which occupies the region $(\,x \ge 0\,).$ To get the unknowns $\pi_{1} ,\,\pi_{2} ,\,\pi_{3}$ the appropriate boundary conditions at * x = 0,* for the considered problem are given by.

i) A thermal boundary relation

24 *θ = 0.*

ii) A mechanical boundary relation

25 $$\sigma_{xx} = - f(y,t) = - f^{*} e^{i \, ay - mt} ,\;\sigma_{xy} = 0,$$

where,$f\,{(}y{,}t{)}$ is an arbitrary function of $y,\,\,t,$ and $\,f^{*}$ is the value of the initial mechanical force. Carrying out the above boundary relations at the surface * x = 0,* we get a group of three equations. After carrying out the inverse of the matrix technique, we have.

26 $$\left( {\begin{array}{*{20}c} \begin{gathered} \pi_{1} \hfill \\ \hfill \\ \end{gathered} \\ \begin{gathered} \pi_{2} \hfill \\ \hfill \\ \end{gathered} \\ {\pi_{3} } \\ \end{array} } \right) = \left( {\begin{array}{*{20}c} \begin{gathered} F_{21} \hfill \\ \hfill \\ \end{gathered} & \begin{gathered} F_{22} \hfill \\ \hfill \\ \end{gathered} & \begin{gathered} F_{23} \hfill \\ \hfill \\ \end{gathered} \\ {F_{31} } & {F_{32} } & {F_{\begin{subarray}{l} 33 \\ \end{subarray} } } \\ {F_{41} } & {F_{42} } & {F_{43} } \\ \end{array} } \right)^{ - 1} \left( {\begin{array}{*{20}c} \begin{gathered} 0 \hfill \\ \hfill \\ \end{gathered} \\ \begin{gathered} - f^{*} \hfill \\ \hfill \\ \end{gathered} \\ 0 \\ \end{array} } \right)$$

We hold the values of the coefficients $\pi_{1} ,\,\pi_{2} ,\,\pi_{3}$.

## Validation and applications

We analyze the numerical values of the physical coefficients to compare outcomes for a nonlocal thermoelastic material with temperature-dependent properties influenced by rotational effect. This analysis is carried out via the thermoelasticity hypothesis based on the MGL theory, incorporating two relaxation times:

$$\lambda_{0} = 7.76\,{\mathrm{x}}10^{10} \,{\mathrm{N}}\,{.}\,{\mathrm{m}}^{{ - {2}}} ,\,\,\,\mu_{0} = 7.78\,{\mathrm{x}}10^{10} \,{\mathrm{N}}\,{.}\,{\mathrm{m}}^{{ - {2}}} ,\,\,\,\,\rho = 8954\,{\mathrm{kg}}\,{.}\,{\mathrm{m}}^{{ - {3}}} ,\,\,\,\,a = 0.5,\,\,\,\,\,T_{0} = 293\,{\mathrm{K,}}\,\,\,\,\,\,C_{E} = 383.3\,\,{\mathrm{J}}\,{\mathrm{.kg}}^{{ - {1}}} {.}\,{\mathrm{K}}^{{ - {1}}} \,,\,\,$$$$\alpha_{t} = 2.78\,{\mathrm{x}}10^{ - 4} K^{ - 1} ,\,\,\,\,\,\,\,\tau_{0} = \,0.7\,{\mathrm{s}},\,\,\,\,\,\,\tau_{1} = \,0.9\,{\mathrm{s}},\,\,\,\,\,\,f^{*} = 0.5{,}\,\,\,\,\,K = 386\,\,{\mathrm{w}}\,{.}\,{\mathrm{m}}^{{ - {1}}} {.}\,{\mathrm{K}}^{{ - {1}}} {\mathrm{.s}}^{{ - 1}} {,}\,\,\,\,\,m = m_{0} + i\xi {,}\,\,\,\,\,m_{0} = - \,\,0.5,\,\,\,\,\,\xi = 0.3,\,$$$$\,y = - \,\,0.5,\,\,\,\,\,\,\,\,\,t = 0.05\,{\mathrm{s}}$$.

**Fig. 2:**
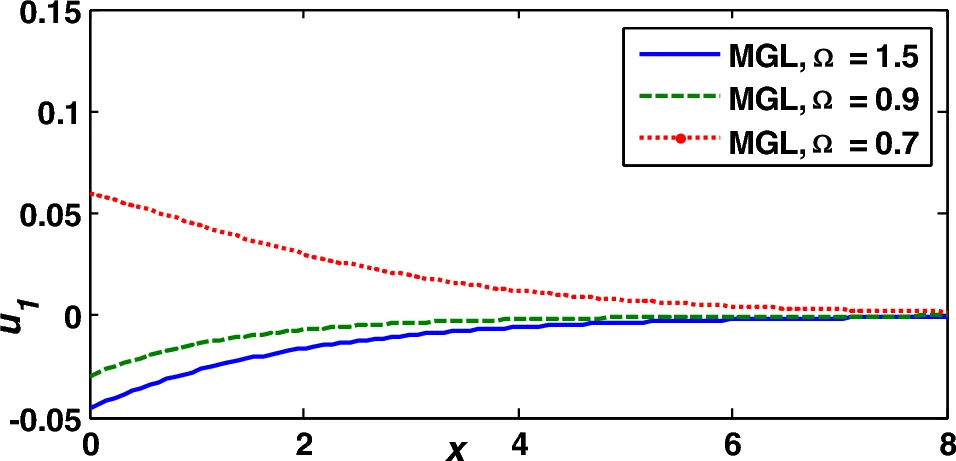
Horizontal displacement distribution $u_{1}$ for variable magnitudes of rotation.

**Fig. 3:**
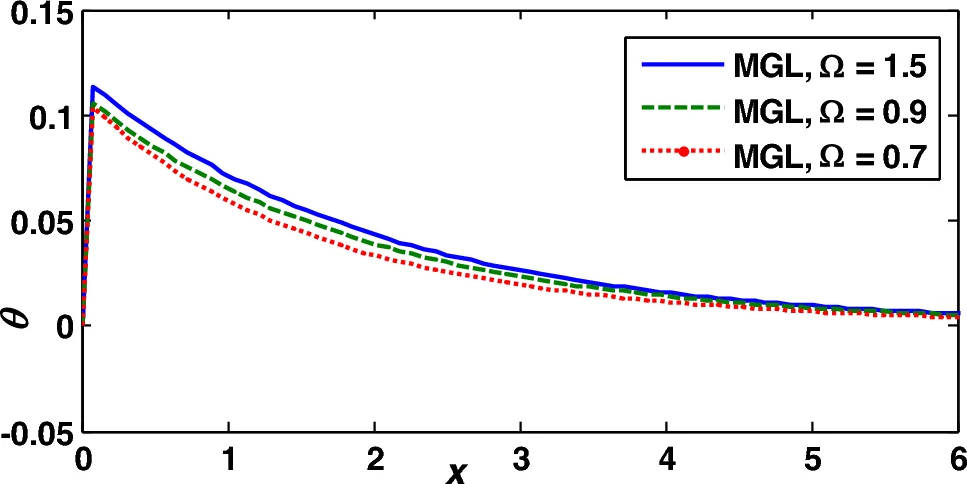
Thermal temperature distribution $\,\theta$ for variable magnitudes of rotation.

**Fig. 4:**
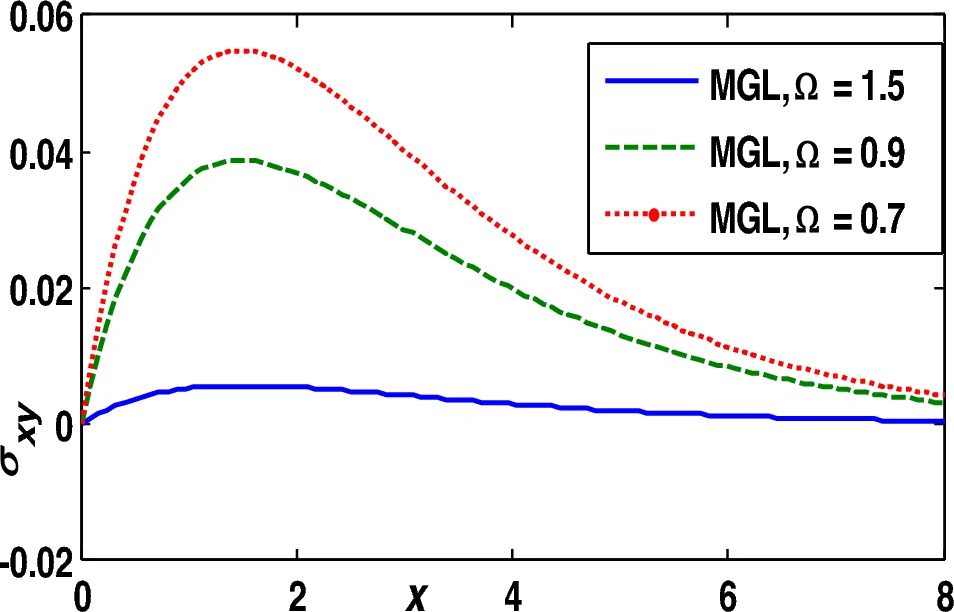
Distribution of stress component $\,\sigma_{xy}$ for variable magnitudes of rotation.

Figures 2, 3, 4 illustrate the distribution of the horizontal displacement $u_{1} ,$ thermodynamic temperature *θ ,*and stress component $\,\sigma_{xy}$ for a nonlocal thermoelastic material with temperature-dependent properties under varying rotation values $\,(\,\,\Omega = 1.5,\,\,0.9,\,\,0.7\,)\,.$Figure 2 illustrates the variation of the displacement component $u_{1}$ along the distance *x* for three values of the rotation parameter $\,(\,\,\Omega = 1.5,\,\,0.9,\,\,0.7\,)\,$ based on the modified Green–Lindsay (MGL) thermoelastic model. The figure clearly demonstrates that the rotation parameter has a significant influence on both the magnitude and the direction of the displacement component. At the boundary (*x* = 0), the displacement assumes its maximum absolute value for each case. For $\,\Omega = 1.5\,$ the displacement is negative with an initial value of approximately − 4.6 × 10^−2^, whereas for $\,\Omega = 0.9\,$ the negative magnitude decreases to about − 2.5 × 10^−2^. In contrast, when the rotation parameter is reduced to $\,\Omega = 0.7\,$ the displacement becomes positive with an initial value close to 6.0 × 10^−2^. This behavior indicates that the rotation parameter not only modifies the amplitude of the displacement but can also reverse its direction. As the distance from the boundary increases, the displacement gradually approaches zero for all rotation values, indicating the attenuation of the thermoelastic disturbance inside the medium. The convergence of all curves beyond approximately *x* = 6 suggests that the influence of rotation is primarily concentrated near the loaded surface, while its effect becomes negligible deeper within the half-space. Furthermore, increasing the rotation parameter from $\,\Omega = 0.7\,$ to $\,\Omega = 1.5\,$ produces a marked reduction in the displacement amplitude and changes its sign, reflecting the strong coupling between rotational motion and thermoelastic wave propagation. This behavior can be attributed to the additional inertial effects introduced by rotation, which alter the stress–displacement interaction and modify the propagation characteristics of the thermoelastic field. Figure 3 illustrates the variation of the temperature *θ* with the distance *x* for three values of the rotation parameter $\,(\,\,\Omega = 1.5,\,\,0.9,\,\,0.7\,)\,$ based on the modified Green–Lindsay (MGL) thermoelastic model. The figure shows that the temperature field is strongly influenced by the rotation parameter. At the boundary (*x* = 0), the temperature reaches its maximum value for all cases, with approximate values of 0.1120, 0.1080, and 0.1030 for $\,\,\Omega = 1.5,\,\,0.9,\,\,0.7\,$, respectively. This indicates that increasing the rotation parameter enhances the peak temperature within the medium. As the distance *x* increases, the temperature decreases monotonically for all values of *Ω*, approaching zero at larger distances from the boundary. This behavior reflects the attenuation of the thermal disturbance due to heat diffusion and thermoelastic coupling. The decay is relatively rapid near the boundary, while it becomes more gradual farther into the half-space. A comparison of the three curves reveals that larger values of the rotation parameter consistently produce higher temperature distributions throughout the medium. Although the differences among the curves are most pronounced in the vicinity of the boundary, they gradually diminish with increasing distance, and the temperature profiles nearly coincide beyond* x* ≈ 5. This observation indicates that the influence of rotation is localized near the heat source and becomes negligible deep inside the material. Overall, the figure demonstrates that the rotation parameter significantly affects the magnitude and spatial distribution of the temperature field. Therefore, rotational effects should be taken into account when analyzing thermoelastic behavior in rotating thermoelastic materials operating under thermal loading. Figure 4 presents the variation of the normal stress component $\,\sigma_{xy}$ with the distance *x* for three values of the rotation parameter $\,(\,\,\Omega = 1.5,\,\,0.9,\,\,0.7\,)\,.$ At the boundary (*x* = 0), the stress is approximately zero for all cases, satisfying the prescribed boundary conditions. As the distance from the boundary increases, $\,\sigma_{xy}$ rises rapidly, reaches a maximum value, and then gradually decreases toward zero as *x* increases. This behavior reflects the generation, propagation, and attenuation of thermoelastic stresses within the medium. The peak stress occurs at approximately *x* ≈ 1.5 for all rotation values, but its magnitude depends strongly on the rotation parameter. For the lowest rotation parameter $\,(\,\Omega = 0.7\,)\,$, the maximum stress is approximately 5.5 × 10^−2^, whereas it decreases to about 3.9 × 10^−2^ for $\,\Omega = 0.9\,$ and further reduces to nearly 6 × 10^−3^ for $\,\Omega = 1.5\,.\,$ These results indicate that increasing the rotation parameter substantially suppresses the stress amplitude throughout the medium. As the distance increases beyond the peak region, all stress curves exhibit a gradual decay toward zero, indicating the attenuation of thermoelastic waves with distance from the loaded boundary. Overall, the figure confirms that the rotation parameter is a key factor controlling the evolution of the stress field in rotating thermoelastic materials. An increase in rotation reduces the stress concentration and modifies the propagation characteristics of thermoelastic waves, highlighting the importance of rotational effects in the design and analysis of rotating engineering structures subjected to thermal loading. The same behavior that has been observed in the literature, by Salah et al.^36^.

**Fig. 5:**
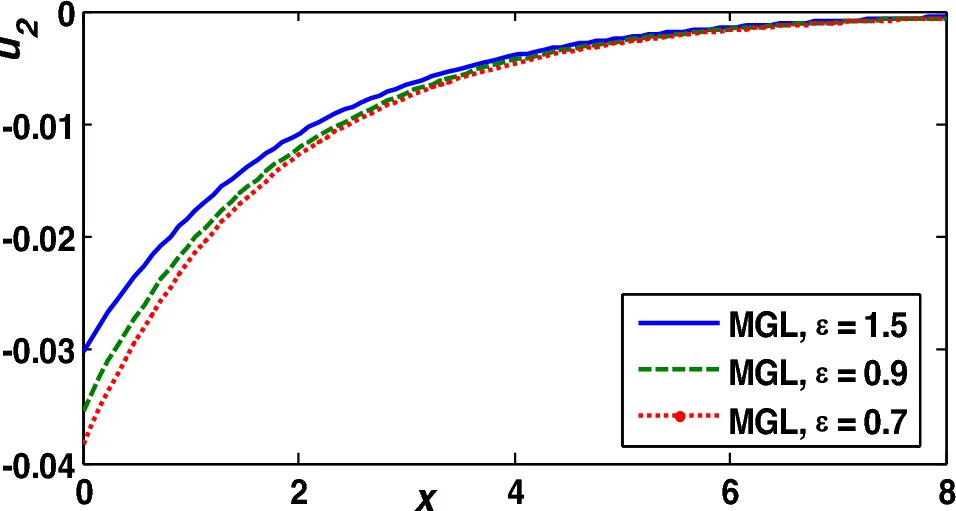
Vertical displacement distribution $u_{2}$ for variable magnitudes of locality parameter.

**Fig. 6:**
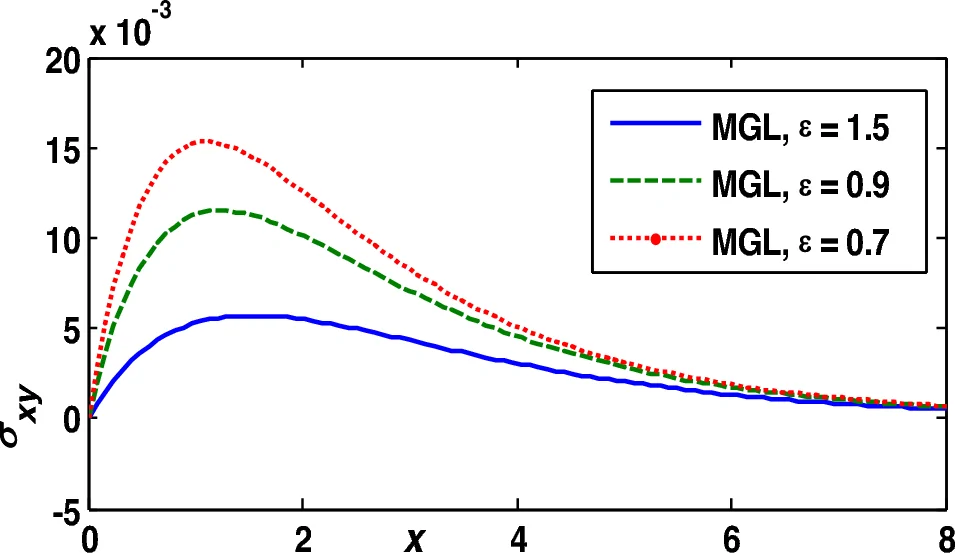
Distribution of stress component $\,\sigma_{xy}$ for variable magnitudes of locality parameter.

Figures 5 and 6 illustrate the vertical displacement $u_{2}$ and the stress component $\sigma_{xy}$ for a nonlocal thermoelastic material with temperature-dependent properties under varying magnitudes of the locality parameter $\,(\,\,\varepsilon = 1.5,\,\,0.9,\,\,0.7\,)\,.$ Figure 5 illustrates the variation of the displacement component $u_{2}$ with the distance *x* for three values of the locality parameter $\,\varepsilon = 1.5,\,\,0.9,\,\,0.7$ within the modified Green–Lindsay (MGL) thermoelastic model. The figure indicates that the displacement component $u_{2}$ is negative throughout the computational domain and gradually approaches zero as the distance from the boundary increases. This behavior reflects the attenuation of the thermoelastic disturbance as it propagates into the half-space. At the boundary (*x* = 0), the magnitude of the displacement is strongly influenced by the locality parameter. For *ε* = 1.5, the displacement is approximately − 3.1 × 10^−2^, while it decreases to about − 3.5 × 10^−2^ for *ε* = 0.9 and reaches nearly − 3.8 × 10^−2^ for *ε* = 0.7. Thus, reducing the locality parameter increases the magnitude of the negative displacement, whereas increasing *ε* suppresses the displacement response. As the distance *x* increases, the three curves rise monotonically toward zero and become almost indistinguishable beyond approximately *x* ≈ 5. This convergence indicates that the influence of the locality parameter is primarily confined to the region close to the loaded boundary, while its effect gradually diminishes deeper within the medium. Increasing *ε* reduces the magnitude of the displacement and promotes a more stable thermoelastic response, emphasizing the importance of accurately selecting this parameter in the analysis and design of advanced composite structures. Figure 6 depicts the variation of the shear stress $\,\sigma_{xy}$ with the distance *x* for different values of the varying values of the locality parameter *ε*. The shear stress exhibits a wave-like behavior, increasing rapidly from the boundary to a maximum value before decaying monotonically with distance. It is observed that reducing *ε* markedly increases the peak value of $\,\sigma_{xy} \,,$ whereas increasing *ε* suppresses the stress response due to the stronger relaxation effect associated with the thermoelastic memory. Although the peak magnitude changes considerably, its position remains nearly constant. At sufficiently large distances, the stress distributions for all values of *ε* approach zero, confirming the attenuation of the thermoelastic wave as it propagates through the rotating fiber-reinforced composite medium. These observations indicate that the locality parameter plays a significant role in controlling the amplitude and attenuation of the induced shear stress. The same behavior that has been observed in the literature, by Said et al.^37^.

**Fig. 7:**
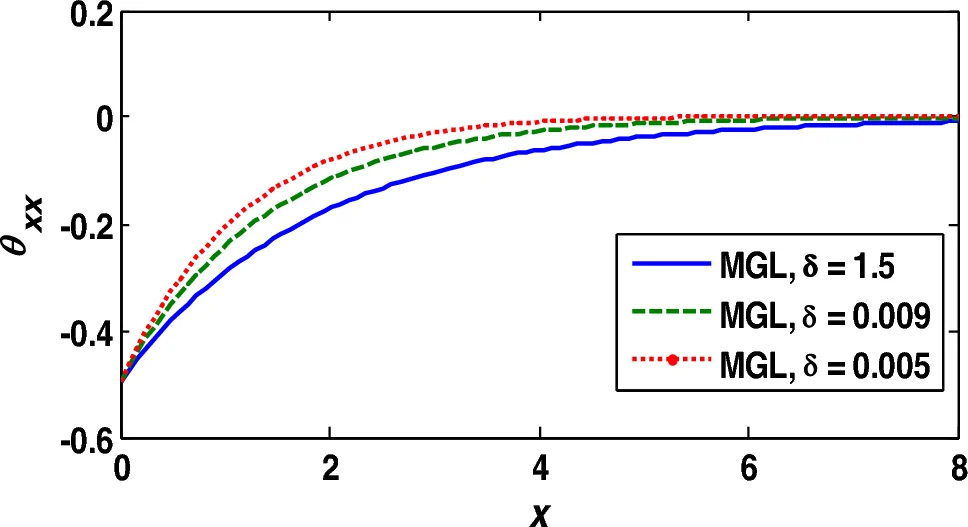
Distribution of stress component $\,\sigma_{xx}$ for variable magnitudes of an empirical material constant.

**Fig. 8:**
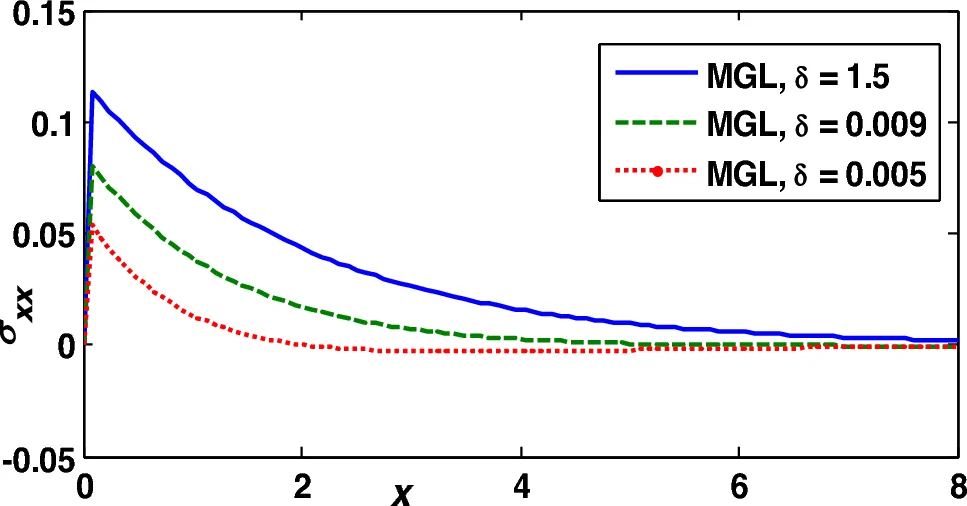
Thermal temperature distribution $\,\theta$ for variable magnitudes of an empirical material constant.

**Fig. 9:**
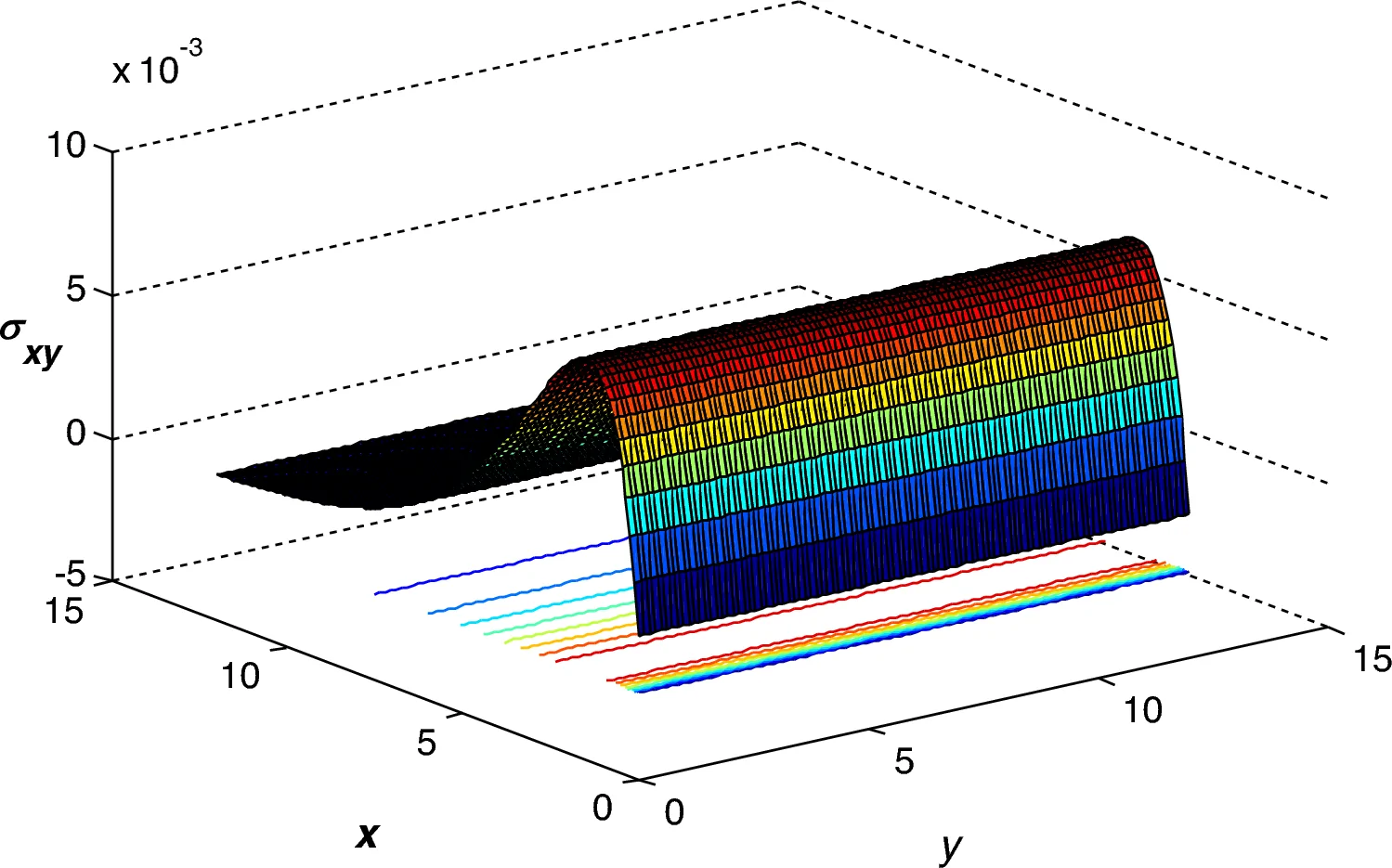
Distribution of stress component $\,\sigma_{xy}$ in three-dimensional.

**Fig. 10:**
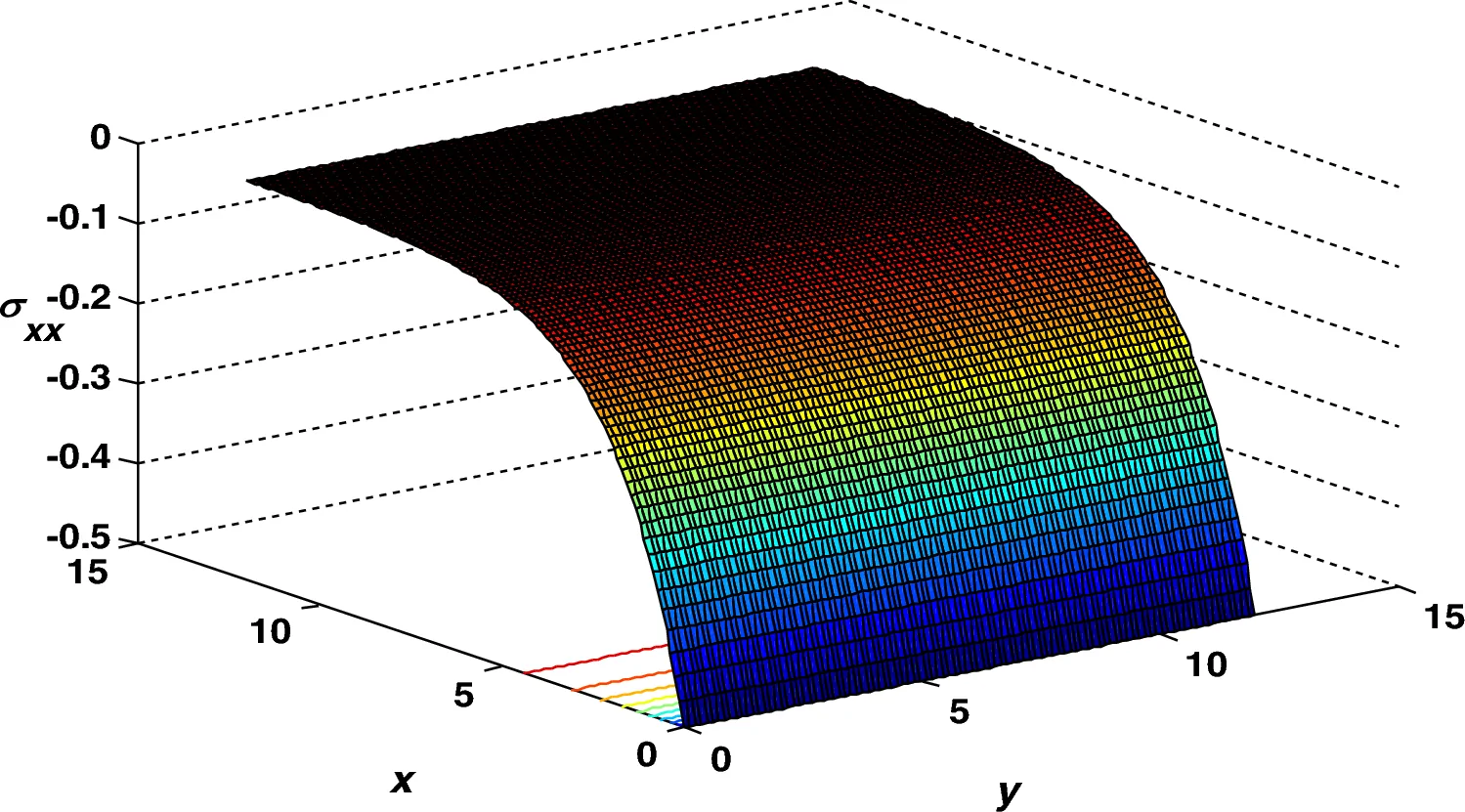
Distribution of stress component $\,\sigma_{xx}$ in three-dimensional.

Figures 7 and 8 display the variation in stress component $\sigma_{xx}$ and thermal temperature distribution *θ* for a nonlocal rotating thermoelastic material, analyzed under different values of an empirical material constant $\,(\,\,\delta = 1.5,\,\,0.009,\,\,0.005\,)\,.$ Figure 7 depicts the effect of the empirical material constant *δ* on the normal stress component $\sigma_{xx} .$ The stress remains negative over the entire spatial domain, indicating a compressive state, and gradually increases toward zero as the distance from the loaded boundary increases due to wave attenuation. It is evident that decreasing *δ* reduces the magnitude of the compressive stress and enhances its decay rate. Consequently, the curve corresponding to* δ* = 0.005 reaches values closest to zero, whereas the largest compressive stresses are obtained for *δ* = 1.5. These findings demonstrate that the empirical material constant governs both the amplitude and attenuation characteristics of the stress field, with lower values promoting faster stress relaxation and higher values sustaining the thermoelastic response over greater distances. Figure 8 depicts the effect of the empirical material constant *δ* on the temperature field *θ .* The temperature decreases monotonically from its maximum value at the heated boundary, demonstrating the propagation and gradual attenuation of the thermal disturbance within the rotating thermoelastic medium. Increasing *δ* enhances the temperature amplitude and extends the thermal penetration depth, whereas decreasing *δ* suppresses the temperature response and increases the attenuation rate. Furthermore, for *δ* = 0.005, the temperature profile exhibits a small negative overshoot before converging to the equilibrium value, indicating the presence of a relaxation-induced wave effect. These results confirm that the memory parameter plays a crucial role in controlling both the intensity and the propagation characteristics of thermal waves in the generalized thermoelastic medium. Figures 9 and 10 provide three-dimensional representations of the non-dimensional physical fields $\sigma_{xx} ,\,\sigma_{xy}$ as a function of displacement $x,\,\,y$ within the framework of the modified Green-Lindsay model. These visualizations play a critical role in understanding how physical quantities vary in correlation with the vertical distance component, thereby offering deeper insights into the spatial dependence of these parameters within the nonlocal thermoelastic framework.

## Conclusion

The analysis clearly indicates that the new model of modified Green-Lindsay thermoelasticity predicts novel behaviors for temperature, displacement, and stresses. The key conclusions derived from the discussion are as follows:

- The nonlocal coefficient has a favorable influence on the distribution of the physical quantities.
- Rotation plays a real role in shaping the fluctuation of physical fields.
- The empirical material constant and vertical distance exert a noteworthy impact on the behavior of physical quantities.
- Specific cases of the modified Green-Lindsay framework include the coupled hypothesis, the Lord-Shulman (L-S) hypothesis, and the Green-Naghdi theory.
- The generalized thermoelastic theory is validated by the observation that physical quantities remain limited to a finite spatial region and gradually diminish to zero with increasing distance, signifying a return to equilibrium.

This framework enables the analysis of nanoscale size effects, ensures finite thermal wave speeds, and resolves nonphysical displacement discontinuities at wave fronts, offering a more comprehensive and realistic representation of material behavior. This study further advances the development of generalized thermoelastic models designed to effectively capture wave propagation and heat conduction phenomena within anisotropic and heterogeneous materials. Although these assumptions enable a clear understanding of the coupled effects of the inclined mechanical load, moving heat source, and rotation on the thermoelastic response, future work may extend the formulation to three-dimensional geometries, functionally graded or anisotropic composites, nonlinear constitutive models, and experimental validation to further enhance the applicability of the proposed model. The findings outlined in this study provide significant value to researchers in material science, physicists, and developers of advanced materials, along with professionals focused on advancing hyperbolic thermoelastic theories.

## Data Availability

The datasets used and/or analyzed during the current study available from the corresponding author on reasonable request.

## List of symbols

$\sigma_{ij}$: Tensor of stress
$\varepsilon_{ij}= e$: Tensor of strain
$$\varepsilon_{kk}$$: Dilatation
* T *: Absolute temperature
$T_{0}$: Temperature of material in its natural state, where $\left| {(T - T_{0} )/T_{0} } \right| < 1,\theta = T - T_{0}$
$\tau_{0} ,\tau_{1} \,$: Thermal relaxation coefficient, $\tau_{1} \ge \tau_{0} \ge 0\,$
$\mu_{0} ,\,\lambda_{0} ,\,\gamma_{0}$: Constants of material
$$u_{i}$$: Displacement components
$$\lambda ,\,\mu$$: Lame's constant
$$\delta_{ij}$$: Kronecker's delta
*K*: Thermal conductivity coefficient’s
*ρ*: Density
$C_{E}$: Specific heat at constant strain
*δ*: An empirical medium coefficient
*i*: $${\mathrm{i}} = \sqrt { - 1}$$
*a*: Wave number
*m*: complex coefficient
$\alpha _{t}$: Linear thermal expansion coefficient $\gamma = \left( {3\lambda + 2\mu } \right)\alpha _{t}$
$\underline{\Omega }$: $= \Omega \,\underline{n} \,,$an angular velocity
$$\underline{\Omega } \,\, \wedge {(}\underline{\,\Omega } \,\,{\mathrm{x}}\,\underline{u} \,{)}$$: Centripetal acceleration
$$2{(}\underline{\Omega } \,{\mathrm{x}}\,\underline{{\dot{u}}} \,{)}$$: Coriolis acceleration
